# A Facile Design of Solution-Phase Based VS_2_ Multifunctional Electrode for Green Energy Harvesting and Storage

**DOI:** 10.3390/nano12030339

**Published:** 2022-01-21

**Authors:** Supriya A. Patil, Iqra Rabani, Sajjad Hussain, Young-Soo Seo, Jongwan Jung, Nabeen K. Shrestha, Hyunsik Im, Hyungsang Kim

**Affiliations:** 1 Department of Nanotechnology and Advanced Materials Engineering, Sejong University, Seoul 05006, Korea; supriyaapatil11@gmail.com (S.A.P.); iqra.rabani@yahoo.com (I.R.); shussainawan@gmail.com (S.H.); ysseo@sejong.ac.kr (Y.-S.S.); jwjung@sejong.ac.kr (J.J.); 2 Division of Physics and Semiconductor Science, Dongguk University, Seoul 04620, Korea; hyunsik7@dongguk.edu (H.I.); hskim@dongguk.edu (H.K.)

**Keywords:** solution-phase, vanadium sulfide, microflower-structure, multifunctional-electrode, green-energy

## Abstract

This work reports the fabrication of vanadium sulfide (VS_2_) microflower via one-step solvo-/hydro-thermal process. The impact of ethylene glycol on the VS_2_ morphology and crystal structure as well as the ensuing influences on electrocatalytic hydrogen evolution reaction (HER) and supercapacitor performance are explored and compared with those of the VS_2_ obtained from the standard pure-aqueous and pure-ethylene glycol solvents. The optimized VS_2_ obtained from the ethylene glycol and water mixed solvents exhibits a highly ordered unique assembly of petals resulting a highly open microflower structure. The electrode based on the optimized VS_2_ and exhibits a promising HER electrocatalysis in 0.5 M H_2_SO_4_ and 1 M KOH electrolytes, attaining a low overpotential of 161 and 197 mV, respectively, at 10 mA.cm^−2^ with a small Tafel slope 83 and 139 mVdec^−1^. In addition, the optimized VS_2_ based electrode exhibits an excellent electrochemical durability over 13 h. Furthermore, the superior VS_2_ electrode based symmetric supercapacitor delivers a specific capacitance of 139 Fg^−1^ at a discharging current density of 0.7 Ag^−1^ and exhibits an enhanced energy density of 15.63 Whkg^−1^ at a power density 0.304 kWkg^−1^. Notably, the device exhibits the capacity retention of 86.8% after 7000 charge/discharge cycles, demonstrating a high stability of the VS_2_ electrode.

## 1. Introduction

Demand on renewable energy is growing impressively day-by-day. However, the heavy reliance on fossil fuels to fulfill the global energy demand has resulted many environmental issues [1]. Consequently, green and efficient energy resources for sustainable energy conversion and storage technologies are highly demandable. Hydrogen (H_2_) is a green energy that may be produced by water-electrolysis. Moreover, the waterlysis can be powered via a renewable energy, and this route of energy production can append a large contribution to achieve the goal on future global carbon neutrality program [2,3]. To produce hydrogen, water electrolysis is commonly catalyzed by noble metal-based catalysts such as those consisting of platinum (Pt) and iridium (Ir). However, these noble metals are rare and are very expensive, resulting the water-electrolysis unaffordable commercially [4,5]. Great efforts have been made to search for noble-metal-free catalysts with high activity and durability. To date, transition metal dichalcogenides (TMDs) [6], metal phosphides [7], MXenes [8] and MOFs [9,10] have been explored as suitable alternatives of Pt for the hydrogen evolution reaction (HER). For example, non-noble metal-based compounds such as MoS_2_, WS_2_, CoS_2_, NiSe_2_, CoSe_2_ [11], amorphous MoSx, VS_2_, CoP, Ni_2_P, FeP, WP, MoP|S, CoPS, NiP_1.93_Se_0.07_, and NiMo alloys have been studied as potential HER electrocatalysts [12,13]. Amongst, Vanadium disulfide (VS_2_) has been emerging as a promising electrocatalyst due to the fact that VS_2_ is a layered 2D-material with high HER catalytic activity both on the basal plane and edge [14,15,16].

On the other hand, another attractive characteristic of VS_2_ is its excellent electrical charge storing ability with a suitable conductivity for charge transport, which is vital for constructing supercapacitors (SCs). Tshifhiwa et al. reported the fabrication of an asymmetric supercapacitor cell based on porous activated carbon material as negative electrode and VS_2_ as positive electrode [17], Similarly, Feng et al. provided a detailed study of Metallic Few-Layered VS_2_ ultrathin nanosheets with less than five S–V–S atomic layers to design practical in-plane supercapacitors for the power sources in advanced ultrathin electronics [18]. Meyer et al. also reported carbon supported VS_2_ nanocomposites possessing the highest possible specific capacitance of 33 Fg^−1^ at a current density of 1 mA which is ascribed to the superior electrical conductivity [19]. SCs are simple electrochemical charge storing devices and have generated substantial research attention due to their facile fabrication process with less environmental concerns, and their high-power density with long cycling stability over the traditional secondary batteries [20]. As a potential SC material, VS_2_ is a hexagonal graphite-like structure composed of S-V-S layers having a large interlayer distance of 5.76 Å, which is suitable to guest Na^+^ and K^+^ ions in the interlayer spacing without a significant structural collapse [17,21]. In addition, the VS_2_ layered structure with suitable electrical conductivity also allows for their rapid electron transport during the charging/discharging cycles [14,21]. Overall, the excellent electrical conductivity, flexible interlayer interactions, high specific surface area with edge exposed atom, and a remarkably resilient, VS_2_ layered structure easily satisfy the requirement basis for developing a promising candidate for electrocatalyst and charge storing electrode-materials [18,22,23].

In this study, we designed a solution phase mediated VS_2_ consisting of microflowered structure, demonstrating the excellent performance on electrocatalytic HER in acidic (0.5 M H_2_SO_4_) and alkaline (1 M KOH) electrolytes together with the electrochemical energy storage capabilities. The optimized VS_2_ electrodes obtained via ethylene glycol added aqueous solvent synthetic route exhibited a highly ordered and smaller microflowered structures distributed uniformly over the larger electrode surface than the one deposited via pure aqueous or ethylene glycol mediated synthesis routes. As a result of their large surface area having abundant active sites and facile charge transport path, the optimized VS_2_ film-based electrodes demonstrated an excellent electrocatalytic HER and electrochemical supercapacitor performance.

## 2. Materials and Methods

All chemicals and reagents were used as obtained without further purification. Sodium orthovanadate (Na_3_VO_4_, 99%), thioacetamide (CH_3_CSNH_2_, 99%), ethylene glycol (HOCH_2_CH_2_OH, 99.8%) and nafion™ perfluorinated resin solution (5 wt.%) were purchased from Sigma Aldrich. Nickel foam (NF,1.6 mm thick) was acquired from Alantum Corporation (Seoul, South Korea). The NF substrate was cut into 1 × 5 cm^2^ pieces and washed for 10–15 min with ultrasonication (LVD-PC 540, 4Science.net, Seoul, Korea) at a frequency of 38.1 kHz for 10 min sequentially in 1 M HCl, deionized water, ethanol, and finally in acetone. The washed NF was dried in air naturally for 24 h at room temperature.

### 2.1. Synthesis of VS_2_ Microflower Structured Powder 

To synthesize VS_2_, 0.1 M sodium orthovanadate and 0.5 M thioacetamide were dissolved in 30 mL DI water by ultrasonic agitation at a frequency of 38.1 kHz for 2 h to obtain a homogeneous solution. The resulting solution was then solvo-/hydro-thermally reacted for 24 h at 160 °C to obtain a black precipitate. The reaction product was naturally cooled to room temperature, then washed several times with DI water and ethanol. After that, the black products were centrifuged at 5000 rpm and finally dried under vacuum in a desiccator. This product is denoted as VS_2_-1 sample. Following a similar procedure, two more VS_2_ powder samples were synthesized for comparison with different solvent composition while keeping all other parameters as they were in the case of VS_2_-1 sample. The VS_2_ sample obtained using pure-ethylene is denoted as VS_2_-2 and the one obtained using 20 mL ethylene glycol and 10 mL DI water mixture solution is named asVS_2_-3 sample.

### 2.2. VS_2_-Based Electrode Fabrication

For catalysis test, the working electrode was prepared by drop casting the catalyst-ink onto a washed NF-substrate. The ink was prepared by mixing 6 mg of the as-synthesized VS_2_ powder in 0.5 mL of a mixture solution obtained from 6.93 mL of DI water-ethanol (3:1 volume ratio) and 5% Nafion (70 µL) solution via ultrasonic agitation at a frequency of 38.1 kHz in normal tap-water for 12 h to form a homogeneous ink. Following the formation of uniform ink, 285 µL was carefully loaded onto a 1 cm^2^ area of the NF substrate. The freshly prepared catalyst film was dried at room temperature for 24 h at 60 °C under vacuum in an oven. The mass loading of the VS_2_ was determined precisely using an inductively coupled plasma mass spectrometry (ICP-MS, NexION^®^ 300D, PerkinElmer, Waltham, MA, USA).

A symmetric device was assembled to study the supercapacitor performance of the as-synthesized VS_2_ based electrodes. For assembly of the symmetric device, VS_2_-1, VS_2_-2, or VS_2_-3 based films on NF-substrate were utilized as positive and negative electrodes and 1 M aqueous solution of KOH was employed as electrolyte. A cellulose membrane was used as a separator, sandwiched between two the symmetric electrodes. The electrodes (VS_2_-1, VS_2_-2, and VS_2_-3) were prepared by mixing the carbon black and polyvinylidene (PVDF) with a mass ratio of 80:10:10, respectively. Then, the mixture was ground by adding a few drops of N-methyl-2-pyrrolidene (NMP) in a mortar until a uniform ink was obtained. The ink was dropped uniformly on the Ni foam and dried in a vacuum oven at 60 °C for 24 h.

### 2.3. Electrochemical Measurements

The multifunctional electrochemical characteristics of the VS_2_-1, VS_2_-2, and VS_2_-3 films on NF-substrates were performed using a three-electrode system (CHI 660D electrochemical instrument). HER experiments were performed in acidic (0.5 M H_2_SO_4_) and alkaline (1 M KOH) electrolytes at a scanning speed of 5 mV∙s^−1^ at room temperature (25 °C). Carbon rods and active ingredients (VS_2_-1, VS_2_-2, and VS_2_-3) coated nickel foams (NF) were used as the counter electrode and working electrode, respectively. The Ag/AgCl was employed as a reference electrode and a cyclic voltammetry (CV) was applied to activate the working electrode for several cycles until constant CV curves were obtained. A linear sweep voltammetry (LSV) was conducted for evaluation of HER and *iR* losses were compensated to the LSV data. 

All the potentials were calibrated to the RHE potential scale using the Equation (1)

*E*_RHE_ = *E*_Ag/AgCl_ + (0.197 + 0.0591pH). (1)

Electrochemical impedance spectra (EIS) were measured in the frequency ranging from 0.1 Hz to 100 kHz.

For the supercapacitor-performance evaluation, cyclic voltammetry (CV), galvanostatic charge/discharge (GCD), and chronopotentiometry (CP) were measured. All electrochemical measurements were carried out using an SP-130 biologic electrochemical workstation at room temperature (25 °C). 

## 3. Results and Discussion

### 3.1. Topographic, Crystal Structure and Chemical Composition Charaterization of VS_2_

**Scheme 1:**
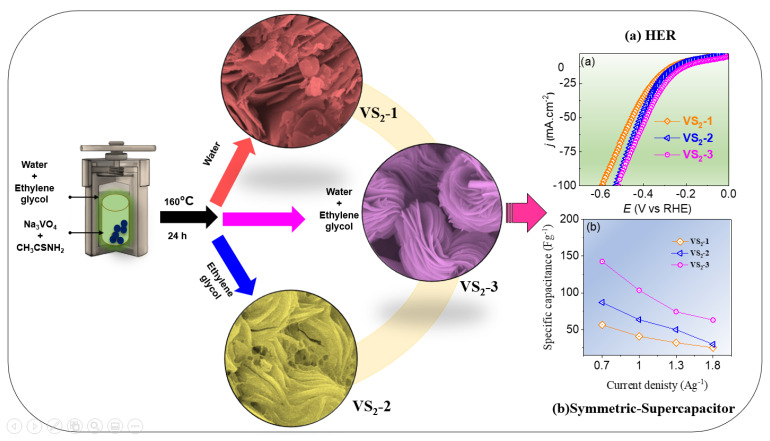
Schematic illustration of the VS_2_ electrodes fabrication and its electrochemical applications, (**a**) Hydrogen evolution and (**b**) Symmetric-Supercapacitor.

**Figure 1:**
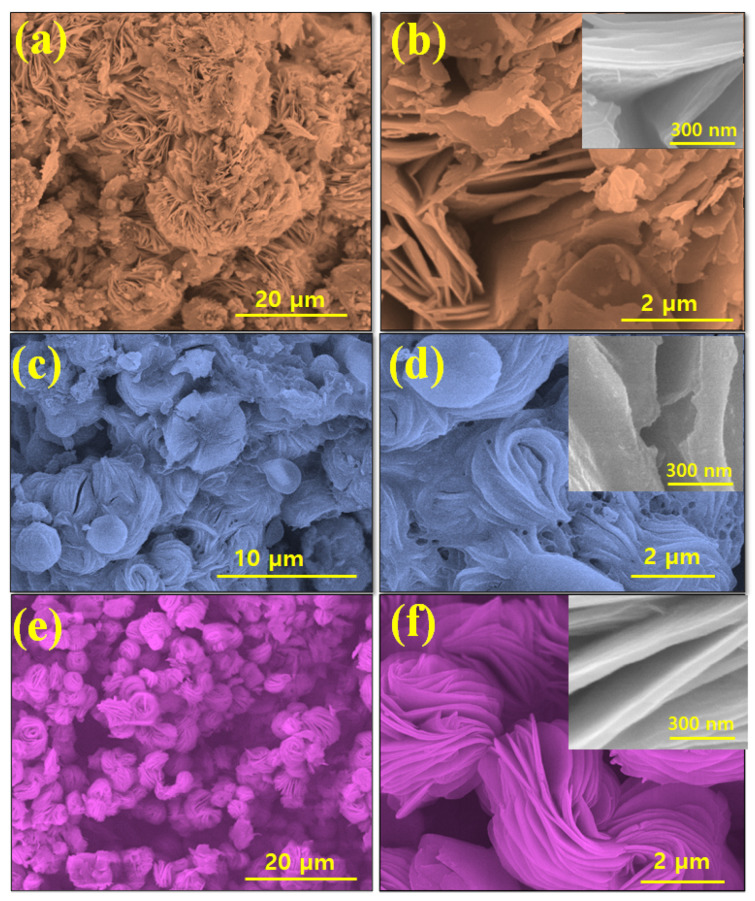
Morphology characterization: FE-SEM images at various magnification of VS_2_ electrodes fabricated using various solvents, (**a**,**b**) water, (**c**,**d**) ethylene glycol, and (**e**,**f**) ethylene glycol + water. (Inset figure shows corresponding high resolution SEM images of VS_2_ electrodes.

The synthetic process of the VS_2_ microflower structured powder is schematically illustrated in Scheme 1. The VS_2_ microflower morphologies (VS_2_-1, VS_2_-2, and VS_2_-3) were obtained via solvo-/hydro-thermal reaction of Na_3_VO_4_·10H_2_O and thioacetamide (C_2_H_5_NS) at 160 °C for 24 h. The morphology of the fabricated VS_2_-1, VS_2_-2, and VS_2_-3 powder was characterized by field-emission scanning electron microscopy (FE-SEM, Hitachi SU8010, Seoul, South Korea) and the resulting images are shown in Figure 1a–f. Figure 1a,b shows randomly oriented nanosheets in a microflowered structure obtained using a pure-aqueous solvent. The diameter of these microstructures lies between 15 and 30 mm and the microstructures look similar to the assembly of flower-petals having 50 nm thick (inset Figure 1b). A similar layered nanosheet or nanoflake-structure of the VS_2_ was reported by hydrothermal synthesis in pure water [17,18,19].

While synthesizing in pure ethylene glycol solvent, the resulting VS_2_ microflowers were agglomerated, and the nanosheets were stacked one above another very closely (Figure 1c,d). A similar VS_2_ microflowers structure has been reported by Chen et al. while synthesizing in propylene glycol and ammonia solution [24]. In contrast, the VS_2_-3 based electrode, which was fabricated from the corresponding power sample synthesized in a mixture solvent of ethylene glycol and water, exhibited uniformly distributed microflowered structures with a diameter of about 1~2 µm (Figure 1e,f). This flower structure was consisted of an assembly of highly ordered unique sheet-like open flower-petal structure with a thickness of ~100 nm, which can be observed at a closer microscopic examination (inset Figure 1f). This highly ordered unique assembly would be beneficial for the improvement of catalytic and supercapacitor performance. It should be noted that the microstructure in this sample was approximately 4 and 15 times smaller than those achieved in a pure-ethylene glycol and pure-water-mediated process, respectively. This is due to the fact that in comparison to the pure water-mediated reaction process, the addition of ethylene glycol lowers the solubility and mobility of the reactants, hindering the crystal formation [25]. As a result, smaller flowers were achieved, which would be beneficial to increase the concentration of active catalytic sites. Furthermore, due to the reductive nature of the alcohol moiety, ethylene glycol can help to minimize the intrinsic defect concentration of the VS_2_ structure, and impurity, such as vanadium oxidation, formation can be prevented during synthesis [26,27]. Remarkably, each nanosheet in VS_2_-3 sample is linked to the next in a highly ordered fashion, generating an open microflower-like structure. This structural feature would be advantageous for free flow of charges between the structures and the electrolyte, thereby enhancing the charge transfer kinetics in electrolysis and supercapacitors. Furthermore, the VS_2_-3 sample contains a consistent distribution of microstructures of comparable size and shape.

**Figure 2:**
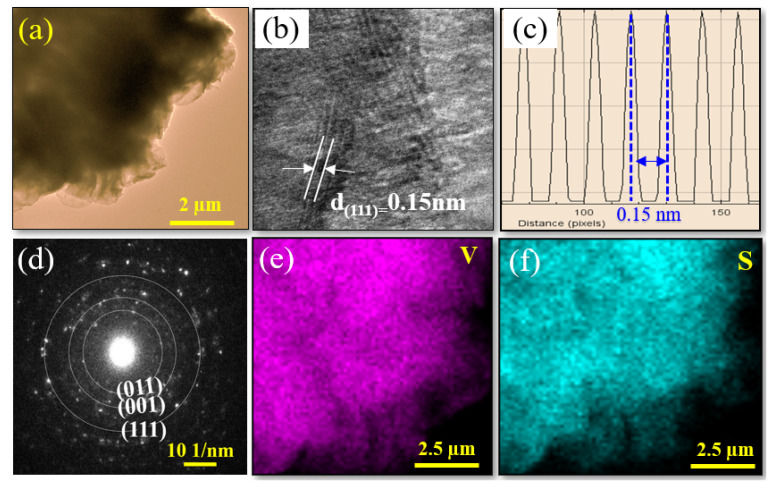
(**a**) TEM images of optimized VS_2_-3 sample (prepared using a combination of ethylene glycol and water solvent), (**b**) High resolution (HR-TEM) images of VS_2_-3 sample, (**c**) intensity peaks image (from FFT) of VS_2_-3 sample calculated using Image*J*, (**d**) Selective area electron diffraction (SAED) patterns exhibiting the crystalline phase, and (**e**,**f**) HAADF-EDS mapping of VS_2_-3 sample.

Transmission electron microscopy (TEM, JEOL-3000F, Seoul, Korea) was also used to demonstrate the formation of the microflowered structure. The TEM depiction of a VS_2_-3 sample obtained from an ethylene glycol/DI water solvent combination is displayed in Figure 2a, which reveals the petal-shaped sheet. The high-resolution TEM image the corresponding inverse FFT spectrum (Figure 2b,c) generated using the ImageJ computer-program (1.8.0_172, Seoul, Korea) indicates a lattice spacing of 0.15 nm, which corresponds to the characteristic (111) crystal-line lattice fringes of VS_2_. Furthermore, the polycrystalline character of the sample is shown by the selected area electron diffraction (SAED) pattern in Figure 2d. The scanning transmission electron microscopy (STEM) image and elemental mapping demonstrate that the V and S elements were distributed consistently throughout the microflowered structure, with no signs of V or S aggregation (Figure 2e,f).

**Figure 3:**
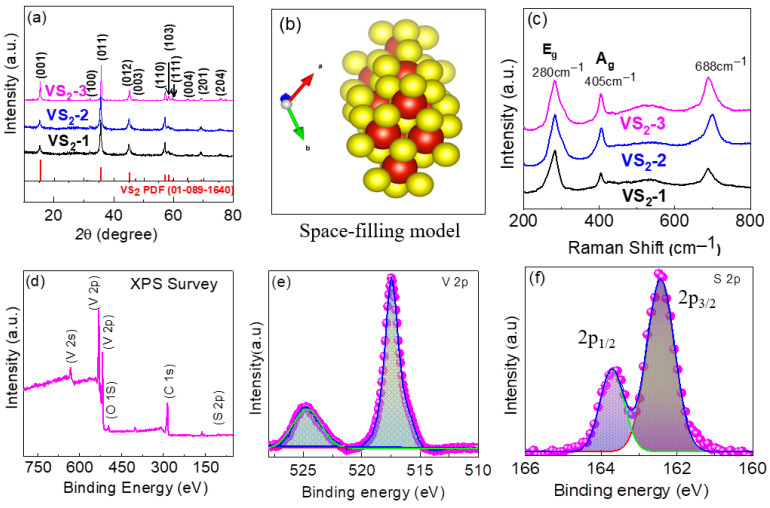
Structural analysis: (**a**) XRD patterns of VS_2_ samples along with standard JCPDS (01-089-1640). (**b**) Space-filling model for VS_2_-3: Red and yellow color balls represent V and S, respectively. (**c**) Raman spectra of the VS_2_-3 electrode, showing three main peaks associated with V-S vibration. (**d**) XPS survey spectrum disclosing the existence of V, S, and O elements. A high-resolution spectrum of the (**e**) V 2p, and (**f**) S 2p of VS_2_-3 electrode. The solid circles and lines represent the experimental data and fitting curves, respectively.

Furthermore, Figure 3a shows the XRD spectra of the as-fabricated VS_2_-1, VS_2_-2, and VS_2_-3 powder samples along with the reference patterns of VS_2_ imported from the JCPDS file no. 01-089-1640. The XRD spectra are well matched to the reference patterns and display various diffraction peaks of the polycrystalline nature. This finding confirms the existence of the hexagonal phase of VS_2_. Based on the standard powder diffraction card, the diffraction peaks (black and blue spectrum) at 2θ = 15.3°, 31.9°, 35.7°, 45.5°, and 47.31° can be indexed to the reflection of (001), (100), (011), (012), and (003) planes of VS_2_, respectively [23]. In addition, the VS_2_-3 sample (magenta color spectrum) also shows two additional different peaks at 58.3° and 59.8 that can be associated with the (103), and (111) planes. These additional VS_2_ phases could further enhance the catalytic site densities in sample VS_2_-3. The corresponding hexagonal crystal phase structure of VS_2_ in a space-filling model is displayed in Figure 3b. Raman analysis was conducted for the VS_2_-1, VS_2_-2, and VS_2_-3 electrodes in the range of 200–800 cm^−1^ as shown in Figure 3c. The three well-defined peaks located at 280 and 405 cm^−1^ can be ascribed to the Eg vibration (S dumbbells librational) mode and the out-of-plane (Ag) vibration mode of the VS_2_, respectively, while the peak at 688 cm^−1^ corresponds to the rocking and stretching vibrations of V–S bonds or their combination [28]. The binding states of the constitutional elements in the optimized VS_2_-3 sample were studied using X-ray photoelectron spectroscopy (XPS), as illustrated in Figure 3d–f. A typical XPS survey spectrum of the VS_2_-3 sample shows that the VS_2_ nanoflower is mostly constituted of V and S, with a small O 1s peak resulting from surface oxidation of VS_2_ in air. The high-resolution spectra of V 2p and S 2p are displayed in Figure 3e,f, respectively. The high-resolution V 2p XPS spectrum can be deconvoluted into a doublet with peaks located at the binding energies of 524.8 eV and 517 eV, respectively, for the V 2p_1/2_ and V 2p_3/2_. This suggests that vanadium in the +4 oxidation state. Similarly, the high-resolution S 2p spectra can be deconvoluted to a doublet with the centers located at the binding energies of 163.6 eV and 162.4 eV, which correspond to the S 2p_1/2_ and S 2p_3/2_, respectively. This reveals the divalent sulfide (S^2−^), and the XPS finding further confirms the VS_2_ (1:2) composition of the sample [29]. In Figure S1, the related EDX spectrum also demonstrates that the V and S stoichiometry is about 1:2, confirming that the synthesized microflower is constituted of VS_2_. Moreover, the VS_2_ particle loading on the NF substrate was determined precisely using ICP-MS, which reveals 3.22 mg cm^−2^, and this sample also demonstrated a V to S atomic ratio of 0.5204, revealing 1:2 stoichiometry of V and S.

### 3.2. HER Performance of VS_2_ Electrodes in Acidic and Alkaline Electrolyte

**Figure 4:**
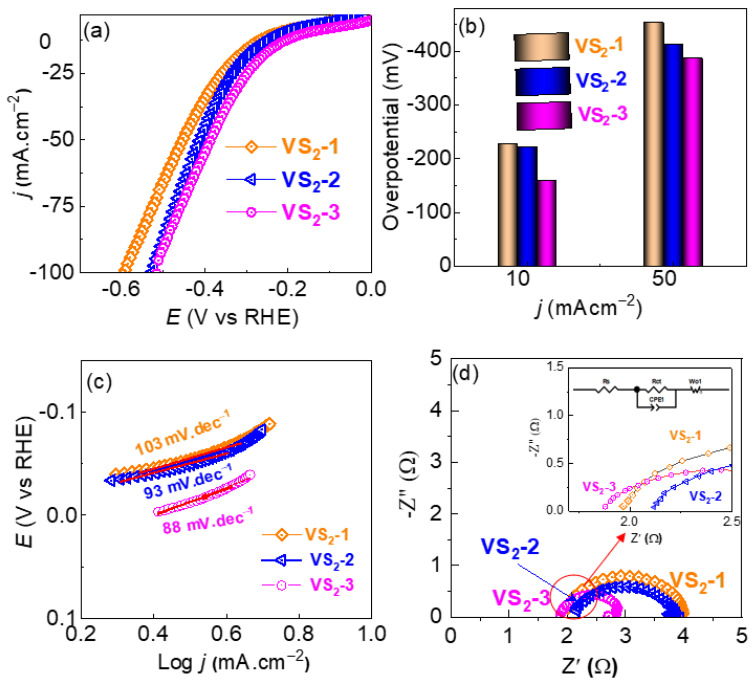
Electrocatalytic performance of the VS_2_ electrodes in a 0.5 M H_2_SO_4_ electrolyte: (**a**) Cathodic LSV polarization curve (*iR* corrected) of VS_2_-1, VS_2_-2, and VS_2_-3 electrodes at a scan rate of 5 mVs^−1^_._ (**b**) HER overpotential vs. current density profiles. (**c**) Corresponding Tafel slopes extracted from the LSVs curves and (**d**) Nyquist plots (inset figure shows the equivalent circuit and magnified view of Nyquist plot at high frequency region).

**Figure 5:**
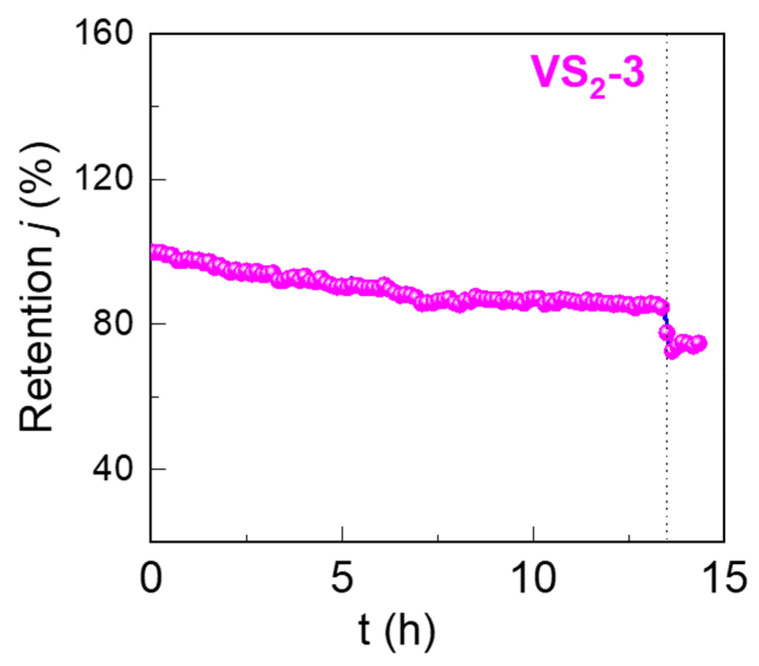
Chronoamperometry profile of the VS_2_-3, showing long-term electrochemical durability for over 13 h during the HER in acidic medium.

The electrocatalytic performances of the VS_2_ based electrodes were assessed for HER using the LSV in a standard three-electrode cell containing 0.5 M H_2_SO_4_ or 1 M KOH aqueous solution as acidic and alkaline electrolyte, respectively. The LSV data were measured at a scan rate of 5 mVs^−1^ and the LSV curves were *iR*-corrected using the solution resistance acquired from the EIS measurement. Figure 4a, shows the polarization curves of the VS_2_-1, VS_2_-2, and VS_2_-3 samples in a 0.5 H_2_SO_4_ aqueous electrolyte. The VS_2_-1 electrode shows the HER overpotential of 227 mV at a current density of 10 mA cm^−2^. The overpotential is lower for the VS_2_-2 electrode obtained from the pure-ethylene glycol-based synthesis route, displaying HER overpotential of 222 at 10 mAcm^−2^. In contrast, the catalyst fabricated utilizing ethylene glycol and water solvent (VS_2_-3) exhibits a relatively lower overpotential of 161 mV at 10 mAcm^−2^. This illustrates that the addition of ethylene into water to synthesize VS_2_-3 catalyst substantially boosted its intrinsic activity. As explained in the earlier section, this is owing to the fact that the addition of ethylene glycol hinders crystal formation and prevents oxide formation when compared to a pure aqueous-mediated process. This led to the highly ordered unique sheet-like open flower-petal microstructure (Figure 1f), thereby boosting the concentration of active catalytic sites. For the performance evaluation, the overpotential was obtained by subtracting the thermodynamic potential of HER (i.e., 0.00 V vs. RHE) from that of the experimentally obtained potential of the working electrode at a given current density. Figure 4b displays a comparative HER overpotential profiles vs. current densities. The VS_2_-3 based electrode, as shown in Figure 4b, has a lower overpotential of 161 and 386 mV at 10, and 50 mAcm^−2^, respectively. This implies that a relatively lower driving energy is needed to drive the HER for the VS_2_-3 sample. In addition, the Tafel plots, presented in Figure 4c, revealed a reasonably low Tafel slope of 88 mV dec^−1^ with a higher exchange current density (*J*_0_) of 7.294 × 10^−5^. This finding reveals the facile HER kinetic for the VS_2_-3 electrode and a smaller amount of driving energy was required towards HER, which is lower than the VS_2_-2 (93 mVdec^−1^, *J*_0_ = 1.640 × 10^−5^) and VS_2_-1 (103 mVdec^−1^, *J*_0_ = 3.863 × 10^−6^) based electrodes, respectively. The charge transport nature of the electrodes was evaluated using electrochemical impedance spectroscopy (EIS), and the resulting Nyquist plot fitted with an analogous circuit is shown in Figure 4d. The relatively low solution resistance (R_s_ = 1.8) and charge-transfer resistance (R_ct_ = 2.41 Ω) experienced by the VS_2_-3 electrode indicates the close contact between the VS_2_-3 active species and NF substrate, and a facile charge transport between the electrolyte and electrode with compared to the VS_2_-1 (R_s_ = 1.9 Ω, R_ct_ = 2.91 Ω) and VS_2_-2 (R_s_ = 2.1 Ω, R_ct_ = 3.08 Ω) based electrodes. The detailed EIS parameters including constant phase element (CPE) and diffusion impedance (Zw) are shown in Table S1. The electrochemical surface area (ECSA) of the catalyst is one of the key factors in electrocatalysis and was computed in terms of the commonly used double-layer charging currents in the non-Faradic regions. The VS_2_-1 (4.0 mFcm^−2^) and VS_2_-2 (4.1 mFcm^−2^) samples showed similar double-layer capacitance while the VS_2_-3 (10 mFcm^−2^) sample exhibited a significantly higher double-layer capacitance (Figure S2). The computed double-layer capacitance is directly related to the ECSA, which is further related directly to the available active electrocatalytic sites. This implies that the VS_2_-1 sample had significantly higher catalytic HER sites compared with the other two samples. As a result, the VS_2_-1 based electrode offered the lower overpotential and faster HER kinetics. Apart from the overpotential and HER kinetic, long-term electrochemical stability of the catalyst-based electrode is also a key factor determining the practical applicability of the catalyst. The long-term electrochemical durability of the VS_2_-3 catalyst-based electrode, which demonstrated a high HER performance amongst other catalyst-based electrodes, was examined via chronoamperometry (*j* vs. t measurement). The HER current density remains fairly stable for a long run over 13 h and has shown the retention of about 85% of the initial current density at the end of 13.43 h. Beyond this, the electrode started to degrade as shown in Figure 5.

**Figure 6:**
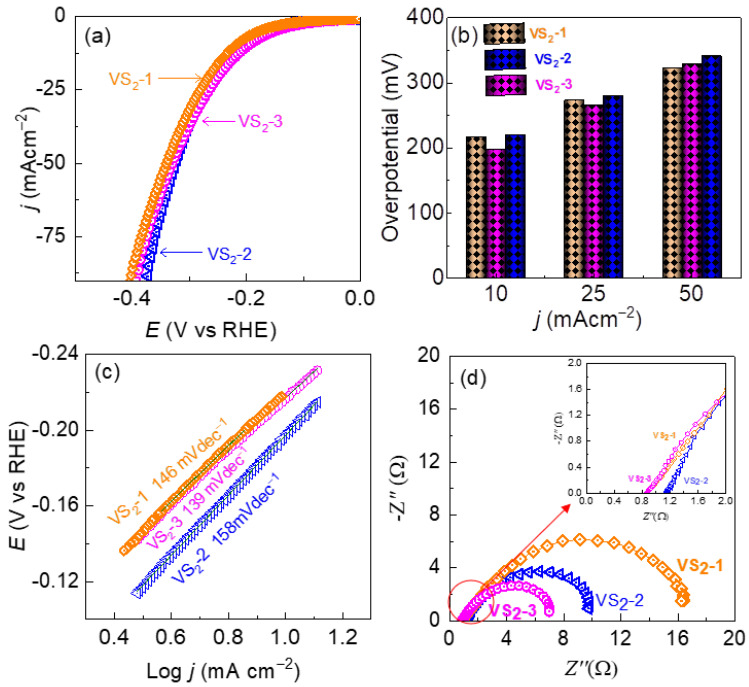
Electrocatalytic performance of the VS_2_ electrodes in a 1 M KOH electrolyte: (**a**) Cathodic LSV polarization curve (*iR* corrected) of VS_2_-1, VS_2_-2, and VS_2_-3 electrodes at a scan rate of 5 mVs^−1^. (**b**) HER overpotential vs. current density profiles. (**c**) Corresponding Tafel slopes extracted from the LSVs curves and (**d**) Nyquist plots (inset figure magnified view of Nyquist plot at high frequency region).

In addition, as shown in Figure 6a, the HER performance of the catalyst was also investigated in 1 M KOH aqueous electrolyte. In line with the HER performance in acidic electrolyte, amongst all, the VS_2_-3 based electrode demonstrated a lower HER overpotential of 197 mV to achieve the current density of −10 mAcm^−2^. As revealed by Figure 6a, this HER activity is relatively lower than that of the VS_2_-1 and VS_2_-2 based electrodes. This finding implies that the solvents used in the synthesis of VS_2_ microstructures play a key role in determining the HER activity via manipulation of microstructure of the catalyst, which can favor for enhancement of the catalytic active sites. Figure 6b depicts a comparative HER overpotential profiles vs. current densities. The VS_2_-3 based electrode, as shown in Figure 6b, has a relatively lower overpotential of 197, 265, and 329 mV at 10, 25, and 50 mAcm^−2^, respectively. Furthermore, the Tafel slope exhibited by the VS_2_-3 based electrode is 139 mVdec^−^^1^, which is lower than that exhibited by the VS_2_-1 (146 mVdec^−1^) and VS_2_-2 (158 mVdec^−1^) based electrodes (Figure 6c). This finding implies that the HER kinetics on the VS_2_-3 electrode is faster than on the other electrodes. Tables S2 and S3 demonstrate a comparison of HER performance of the VS_2_-3/NF electrode with other previously published representative transition metal-based electrocatalysts. To further support this finding on HER kinetics, EIS was measured in 1 M KOH electrolyte, and the resulting Nyquist plot is shown in Figure 6d. In comparison to the VS_2_-1 and VS_2_-2 based electrodes, the VS_2_-3 based electrode exhibited a relatively smaller charge-transfer resistance (R_ct_) as shown by the smaller semicircle in Figure 6d. This implies the facile charge transfer between the VS_2_-3 based electrode and electrolyte indicating the faster HER kinetic.

### 3.3. Supercapacitor Performance of VS_2_ in Symmetric Device-Configuration

**Figure 7:**
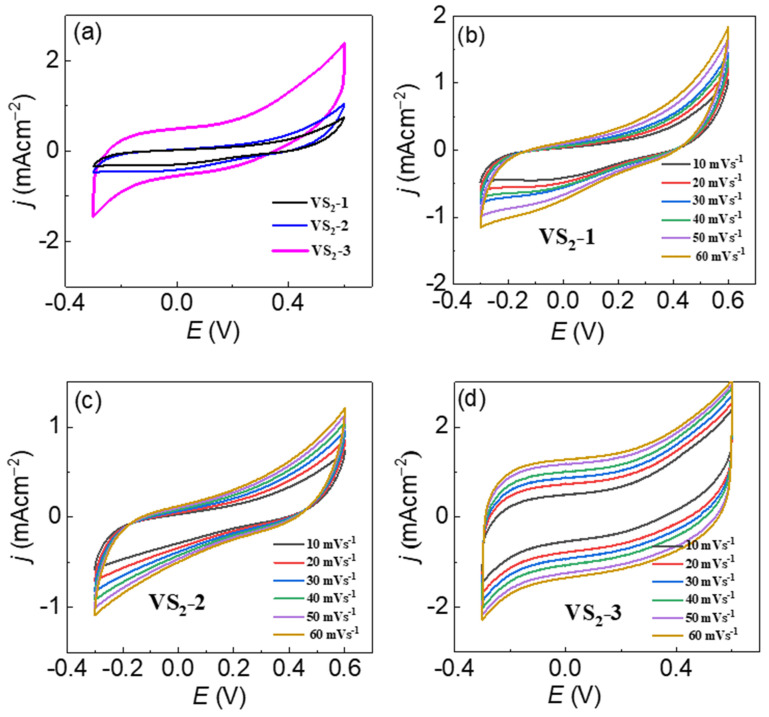
Symmetric supercapacitor-based CV performance: (**a**) CV curve for VS_2_-1, VS_2_-2 and VS_2_-3 at 10 mVs^−1^ of scan rate, (**b**–**d**) CV curve for the VS_2_-1, VS_2_-2 and VS_2_-3 at the 10–60 mVs^−1^ of scan rate.

A symmetric device is employed for the evaluation of the electrochemical performance of VS_2_ electrodes. For this, the as-prepared electrodes were utilized as cathode and anode with 1 M KOH as electrolyte. Cyclic voltammetry (CV) was examined at the voltage ranging between −0.3 to 0.6 V, as shown in Figure 7a–d. The CV curves for the device prepared from the VS_2_-1, VS_2_-2, VS_2_-3 based electrodes are depicted in Figure 7a. The VS_2_-3 electrode-based device clearly exhibited a high current response which was an about 2-folds integral area compared with the VS_2_-1 and VS_2_-2 electrode-based devices. The CV curves of all the VS_2_ based devices exhibited a notably similar shape resembling to the rectangular nature of an electric double layer capacitor (EDLC). However. It should be noted that the CV curves are not ideally rectangular. Although, no distinctive redox peaks in the CV curves can be observed, the curves are, in fact, deviated from the pure-EDLC derived ideal rectangular shape. This indicates the possibility of occurrence of reversible redox reactions within the voltage window under study. In general, the charge storage mechanism can be classified into the diffusion-controlled faradaic components originated from the pure redox reactions taking place by the diffusion of active ions from electrolyte to the electrode, surface-bound rapid faradaic components originated from the redox reaction of the surface adsorbed active ions on the electrode surface, and the surface-bound rapid non-faradaic components resulting from the material’s EDLC behavior [30,31].

Further, the CV curves at various sweep rates (10 mVs^−1^ to 60 mVs^−1^) are recorded in Figure 7b–d. Based on the highly ordered and open petal assembled microflower-like structures of VS_2_ in VS_2_-3 sample, a rapid to-and-fro trafficking of K^+^ ions facilitated by the 2D multi-layered VS_2_ nanosheets can be assumed in the VS_2_-3 based symmetric device. As a consequence, the CV curve of the VS_2_-3 based symmetric device maintains a similar shape even at the higher scan rates such as 60 mVs^−1^, indicating the excellent reversibility of the symmetric device. In contrast, the VS_2_-1 and VS_2_-2 based symmetric devices exhibited the CV curves deviated largely from the rectangular shape, showing severe polarization when the scan rate was increased from 10 to 60 mVs^−1^. The deviation in the shape of CV curves is attributed to the redox reaction across the electrode-electrolyte interface, which corresponds to the contribution from the typical characteristics of the pseudo-capacitance. Notably, when the CV loops were repeated for 100 cycles at the 10 mVs^−1^, unperturbed shape of the CV curve even after a colossal number of cycles were obtained in the VS_2_-3 based symmetric device, exhibiting an about of 98% cyclic retentions. This preliminary finding reveals a long-term stability and higher rate capability of the VS_2_-3 based symmetric device.

**Figure 8:**
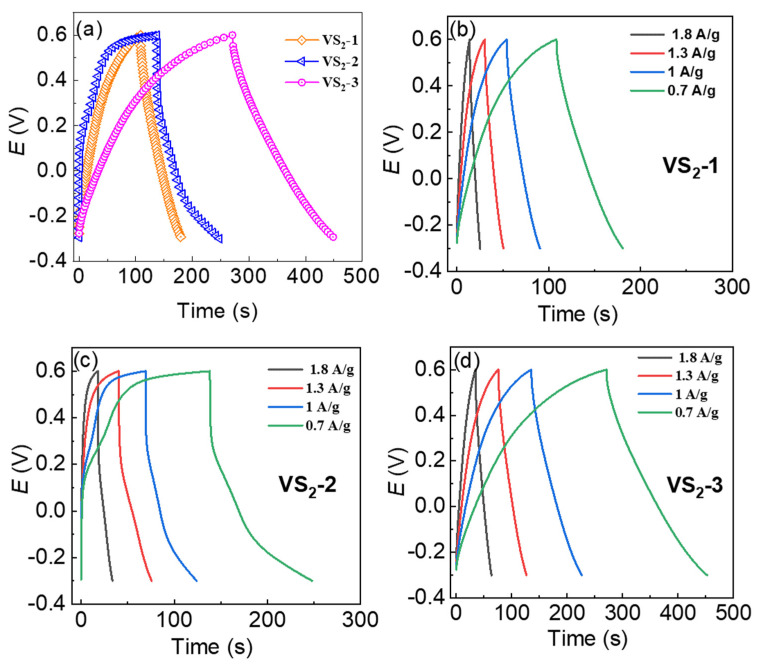
Symmetric supercapacitor-based GCD behavior: (**a**) GCD curve at the 0.7 Ag^−1^ of current density for VS_2_-1, VS_2_-2 and VS_2_-3, (**b**–**d**) GCD curves for the VS_2_-1, VS_2_-2 and VS_2_-3 at the 0.7–1.8 Ag^−1^.

**Figure 9:**
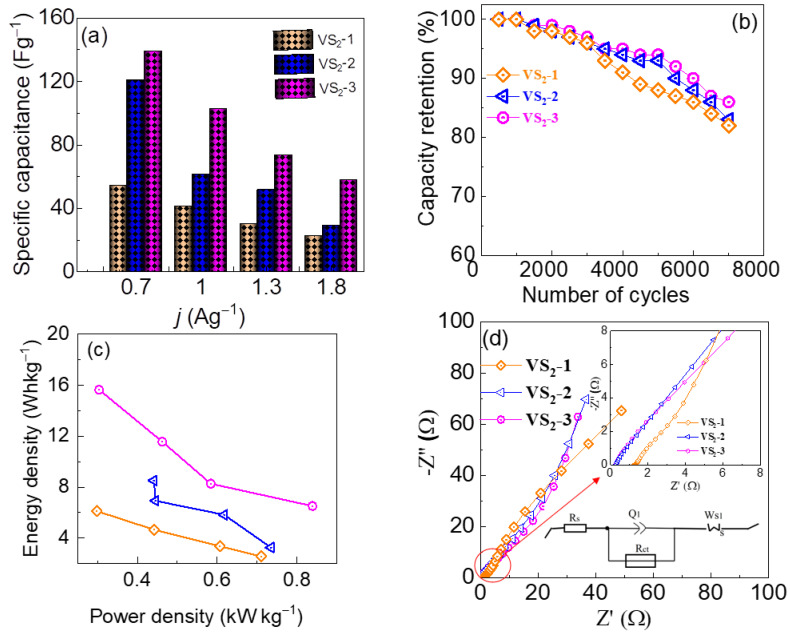
Symmetric supercapacitor performance: (**a**) specific capacitance against at different current densities, (**b**) cycling stability at 0.7 Ag^−1^ of current density, (**c**) Ragone lines and (**d**) Nyquist plot for VS_2_-1, VS_2_-2 and VS_2_-3, respectively (Inset figure shows the corresponding equivalent circuit and magnified view of Nyquist plot at high frequency region).

For in-depth evaluation of the device performance, galvanostatic charging discharging (GCD) has been analyzed at 0.7 Ag^−1^ and the results are presented in Figure 8a. The VS_2_-3 based symmetric device exhibited longest discharging time compared with the others, thereby leading to the higher specific capacitance (Cs), which is consistent with the CV curves. The symmetric device shows smaller *iR* drops particularly at the lower current density of 0.7 Ag^−1^, implying the low internal resistance of the symmetric device. Moreover, the GCD curves for the VS_2_-1, VS_2_-2 and VS_2_-3 based devices were examined at the various current densities, and are depicted in Figure 8b–d. Here, Cs was determined through GCD curve using the following Equation (2).

(2) $$C_{s}=\frac{I_{dis}\times \Delta t_{dis}}{\Delta V}$$

wherein, *I_dis_* is the discharging current density, ∆*t_dis_* is the discharging time, and ∆*V* is the voltage window. The VS_2_-3 symmetric device shows *Cs* of 139 Fg^−1^ which is higher than that of VS_2_-2 (120 Fg^−1^) and VS_2_-1 (54 Fg^−1^) at the discharging current density of 0.7 Ag^−1^ (Figure 9a). The long-term stability of the symmetric device is a key parameter for practical application of the supercapacitors, which was investigated by the repeated GCD measurements at a current density of 0.7 Ag^−1^ for 7000 cycles, as presented in Figure 9b. The capacity retention was almost 100% at the end of 1000 cycles, beyond which it decreased gradually. Notably, the VS_2_-3 based symmetric device showed remarkable capacity retentions of 86% even at the end of 7000 cycles revealing the capacitance degradation of only about 14%, which is lower than that of VS_2_-2 (17%) and VS_2_-1 (18%) based devices. 

The energy and power density of the symmetric devices were determined utilizing the following equation:

(3) $$E=\frac{C_{s}\times \Delta V^{2}}{2}$$

(4) $$P=\frac{E}{\Delta t_{dis}}$$

where, *E* and *P* are energy and power density, respectively. The energy density as a function of power density of the symmetric VS_2_ based devices is shown in Figure 9c. The VS_2_-3 based device exhibited a maximum energy density of 15 Whkg^−1^ at a power density of 0.304 kWkg^−1^. Furthermore, EIS was measured to investigate the charge transfer behaviors of the VS_2_-1, VS_2_-2, and VS_2_-3 electrodes in the frequency ranging from 100 mHz to 200 kHz under an open circuit potential (Figure 9d). The variables *R_s_* and *R_ct_* represent the electrochemical system resistance (i.e., all resistances including electrolyte ionic resistance, substrate intrinsic resistance, and contact resistance between the active material and current collector) and charge transfer resistance, respectively. The *R_s_* and *R_ct_* of the VS_2_-3 device-based electrodes are smaller than those of VS_2_-1, VS_2_-2 based devices (Table S4), indicating that the VS_2_-3 based material in the device transport current fluently to the current collector than the other VS_2_ materials. This EIS data supports the higher performance of the VS_2_-3 based symmetric device. 

## 4. Conclusions

A solvo-/hydro-thermal route was employed to design a solution-phase mediated layered VS_2_ microflower structured materials in a mixture of ethylene glycol and water as the reaction media. The optimized VS_2_-3 based electrode obtained from the ethylene glycol and water mixed solvent-mediated synthesis route demonstrated a high catalytic performance for HER in both acidic and alkaline electrolytes containing 0.5 M H_2_SO_4_ or 1 M KOH. This finding demonstrates the ability of the VS_2_ to catalyze HER in a wide pH range of electrolyte. In addition, the optimized VS_2_-3 electrode-based symmetrical supercapacitor exhibited a specific capacitance of 139 Fg^−1^ with an energy density of 15 Whkg^−1^. Moreover, the device showed an excellent capacity retention of 86.8% of the initial value at the end of 7000 charge/discharge cycles, demonstrating a high stability of the VS_2_ electrode. The strong electrocatalytic and supercapacitor performance is attributed to the crystalline microflower structure of VS_2_ consisting of highly ordered nanosheet layers. This study proposes a straightforward construction technique for green hydrogen energy production and green energy storage devices which can contribute to the future global carbon neutrality program.

## Data Availability

Data can be available upon request from the authors.

## Supplementary Materials

The following supporting information can be downloaded at: https://www.mdpi.com/article/10.3390/nano12030339/s1, Figure S1: Energy-dispersive X-ray spectroscopy (EDX), Figure S2: (a–c) Cyclic voltammetry (CV) curves of the VS_2_-1, VS_2_-2, VS_2_-3 electrodes in the non-faradic region, and (d) their corresponding scan rate versus current density plots of the electrodes. Table S1: EIS parameters extracted from Nyquist plots of the VS_2_ samples. Table S2: Comparison of HER performance of the VS_2_-3 catalyst with the recently reported transition metal-based metal catalysts in acidic (0.5 M H_2_SO_4_) electrolyte. Table S3: Comparison of HER performance of the VS_2_-3 catalyst with the recently reported transition metal-based metal catalysts in alkaline (1M KOH) electrolyte. Table S4: EIS parameters extracted from Nyquist plots of the VS_2_ samples.
