# Supplementary material for: A Facile Design of Solution-Phase Based VS2 Multifunctional Electrode for Green Energy Harvesting and Storage
Source: Nanomaterials (Basel). 2022 Jan 21;12(3):339. doi: 10.3390/nano12030339 (PMC8839757; doi:10.3390/nano12030339)
Supplement: Supplementary file 1 [file nanomaterials-12-00339-s001.zip › nanomaterials-1548800-supplementary.pdf]

Supplemaentary Materials

A Facile Design of Solution-Phase Based VS_2 Multifunctional Electrode for Green Energy Harvesting and Storage

Supriya A. Patil [1], Iqra Rabani [1], Sajjad Hussain [1], Young-Soo Seo [1], Jongwan Jung [1], Nabeen K. Shrestha [2,*], Hyunsik Im [2] and Hyungsang Kim [2]

[1] Department of Nanotechnology and Advanced Materials Engineering, Sejong University, Seoul 05006, Korea; supriyaapatil11@gmail.com (S.A.P.); iqra.rabani@yahoo.com (I.R.); shussainawan@gmail.com (S.H.); ysseo@sejong.ac.kr (Y.-S.S.); jwjung@sejong.ac.kr (J.J.)
[2] Division of Physics and Semiconductor Science, Dongguk University, Seoul 04620, Korea; hyunsik7@dongguk.edu (H.I.); hskim@dongguk.edu (H.K.)
* Correspondence: nabeenkshrestha@hotmail.com

Citation: Patil, S.A.; Rabani, I.; Hussain, S.; Seo, Y.-S.; Jung, J.; Shrestha, N.K.; Im, H.; Kim, H. A Facile Design of Solution-Phase Based VS_2 Multifunctional Electrode for Green Energy Harvesting and Storage. *Nanomaterials* **2022**, *12*, x. https://doi.org/10.3390/nano12030339

Academic Editor: Barbara Ballarin

Received: 25 December 2021
Accepted: 19 January 2022
Published: 21 January 2022

Publisher's Note: MDPI stays neutral with regard to jurisdictional claims in published maps and institutional affiliations.

Copyright: © 2022 by the authors. Licensee MDPI, Basel, Switzerland. This article is an open access article distributed under the terms and conditions of the Creative Commons Attribution (CC BY) license (https://creativecommons.org/licenses/by/4.0/).

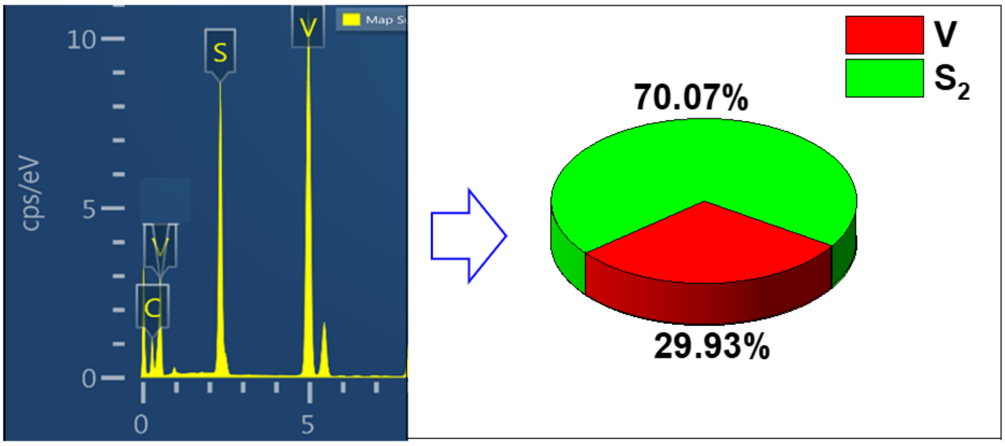

Figure S1. Energy-dispersive X-ray spectroscopy (EDX) analysis spectrum.

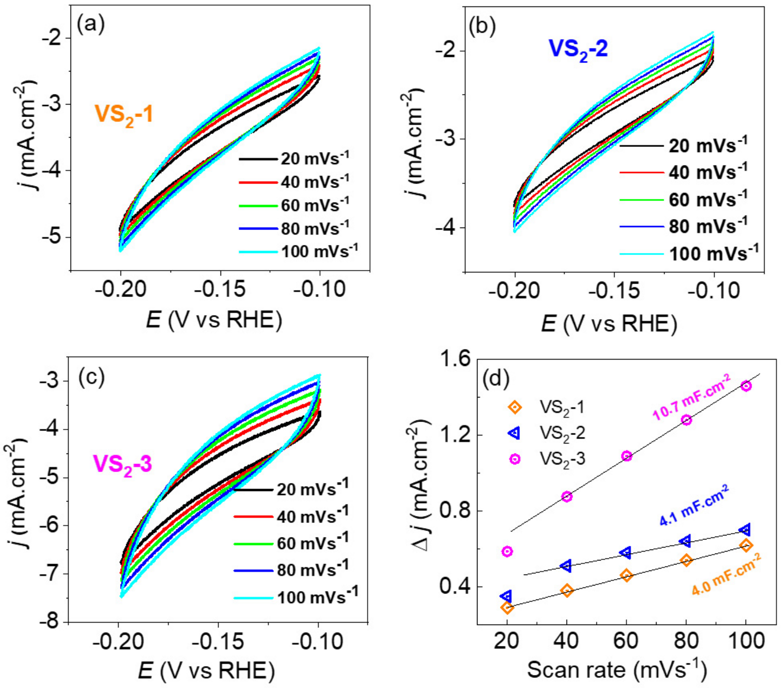

Figure S2. (a-c) Cyclic voltammetry (CV) curves of the VS_2-1, VS_2-2, VS_2-3 electrodes in the non-faradic region, and (d) their corresponding scan rate versus current density plots of the electrodes.

Table S1. EIS parameters extracted from Nyquist plots of the VS_2 samples.

Name	H_2O(S1)	EG(S2)	H_2O+EG (S3)
Rs(Ω)	1.980	2.102	1.870
Rct (Ω)	2.910	3.080	2.410
CPE(μF)	0.710	0.663	0.618
Z_W (Ω)	0.028	0.023	0.021

Table S2. Comparison of HER performance of the VS_2 -3 catalyst with the recently reported transition metal-based metal catalysts in acidic (0.5 M H_2SO_4) electrolyte.

No	Catalyst name	Electrolyte	Overpotential (mV) @ 10mAcm^{-2}	Reference
	**VS_2 -3**	0.5 M H_2SO_4	161	This work
1	SnS_2/NF	0.5 M H_2SO_4	232	*J Hazard Mater*, 417 (2021), p. 126105
2	VS4/rGO	0.1 M H_2SO_4	210	Dalton Trans., 2018,47, 13792-13799
3	CoS_2/rGO	0.5 M H_2SO_4	150	Nano Convergence, 2016, 3:5.
4	NiS_2/rGO	0.5 M H_2SO_4	200	Catalysis Communications, 2016, 85, 26
5	Annealed WS_2/CC	0.5 M H_2SO_4	250	J. Mater. Chem. A, 2015, 3, 131
6	VS_2/ZnS/CdS	0.5 M H_2SO_4	86	Nano Convergence (2016) 3:5
7	VS_2@MoS_2	0.5 M H_2SO_4	177	ACS Appl. Mater. Interfaces 2017, 9, 42139–42148
8	$V_{0.09}Mo_{0.91}S_2$	0.5 M H_2SO_4	240	Nanoscale 2014, 6, 8359–8367

Table S3. Comparison of HER performance of the VS_2 -3 catalyst with the recently reported transition metal-based metal catalysts in alkaline (1M KOH) electrolyte.

No	Catalyst name	Electrolyte	Overpotential (mV) @ 10mAcm^{-2}	Reference
	**VS_2 -3**	1M KOH	197	This work
1	VO-S/NF	1M KOH	165	*Applied Surface Science 423 (2017) 1090–1096*
2	Ni@C/Ni foam	1M KOH	270	*J. Mater. Chem. A* 4 (2016) 7297-7304.
3	$NiCo_2S_4$/carbon cloth	1M KOH	305	*Nanoscale* 7 (2015) 15122-15126.
4	NiFe layered double hydroxides/Ni foam	1M KOH	210	*Science* 345 (2014) 1593-1596.
5	NiFeS/NF	1M KOH	180	*J. Mater. Chem. A* 4 (2016) 16394-16402.

Table S4. EIS parameters extracted from Nyquist plots of the VS_2 samples.

**Name**	**R_s(Ω)**	**R_{ct}(Ω)**
VS_2-1	1.12	1.45
VS_2-2	0.46	1.12
VS_2-3	0.24	0.945
